# Differential antiviral activities of respiratory syncytial virus (RSV) inhibitors in human airway epithelium

**DOI:** 10.1093/jac/dky089

**Published:** 2018-03-27

**Authors:** Carmen Mirabelli, Martine Jaspers, Mieke Boon, Mark Jorissen, Mohamed Koukni, Dorothée Bardiot, Patrick Chaltin, Arnaud Marchand, Johan Neyts, Dirk Jochmans

**Affiliations:** 1 Laboratory of Virology and Chemotherapy, Department of Microbiology and Immunology, Rega Institute for Medical Research, KU Leuven, B-3000 Leuven, Belgium; 2 Research Group Oto-Rhino-Laryngology, KU Leuven and Leuven University Hospitals, B-3000 Leuven, Belgium; 3 Department of Pediatrics, Pediatric Pulmonology, University Hospital Leuven, B-3000 Leuven, Belgium; 4 Department of Development and Regeneration, Organ Systems, KU Leuven, B-3000 Leuven, Belgium; 5 Cistim Leuven vzw, Bioincubator 2, Gaston Geenslaan 2, 3001 Leuven, Belgium; 6 Center for Drug Design and Development (CD3), KU Leuven R&D, Waaistraat 6, B-3000 Leuven, Belgium

## Abstract

**Objectives:**

We report the use of reconstituted 3D human airway epithelium cells (HuAECs) of bronchial origin in an air–liquid interface to study respiratory syncytial virus (RSV) infection and to assess the efficacy of RSV inhibitors in (pre-)clinical development.

**Methods:**

HuAECs were infected with RSV-A Long strain (0.01 CCID_50_/cell, where CCID_50_ represents 50% cell culture infectious dose in HEp2 cells) on the apical compartment of the culture. At the time of infection or at 1 or 3 days post-infection, selected inhibitors were added and refreshed daily on the basal compartment of the culture. Viral shedding was followed up by apical washes collected daily and quantifying viral RNA by RT-qPCR.

**Results:**

RSV-A replicates efficiently in HuAECs and viral RNA is shed for weeks after infection. RSV infection reduces the ciliary beat frequency of the ciliated cells as of 4 days post-infection, with complete ciliary dyskinesia observed by day 10. Treatment with RSV fusion inhibitors resulted in an antiviral effect only when added at the time of infection. In contrast, the use of replication inhibitors (both nucleoside and non-nucleoside) elicited a marked antiviral effect even when the start of treatment was delayed until 1 day or even 3 days after infection. Levels of the inflammation marker RANTES (mRNA) increased ∼200-fold in infected, untreated cultures (at 3 weeks post-infection), but levels were comparable to those of uninfected cultures in the presence of PC786, an RSV replication inhibitor, suggesting that an efficient antiviral treatment might inhibit virus-induced inflammation in this model.

**Conclusions:**

Overall, HuAECs offer a robust and physiologically relevant model to study RSV replication and to assess the efficacy of antiviral compounds.

## Introduction

The human respiratory syncytial virus (RSV) is worldwide the most prevalent viral pathogen associated with acute lower respiratory infection (ALRI) in infants and children.^1^ Based on data collected in 2015, an estimated 33 million episodes of RSV ALRI resulted in ∼3.2 million hospital admissions and 59 600 in-hospital deaths in children younger than 5 years, of which 27 300 occurred in children younger than 6 months.^2^ RSV also causes significant disease in the elderly, as well as in immunocompromised patients and transplant recipients.^3^ Currently, infected patients mainly receive symptomatic treatment and high-risk young paediatric patients (premature, with congenital cardiac abnormality or chronic lung disease) receive prophylactic treatment with the monoclonal antibody palivizumab.^4^ Fourteen trials with RSV vaccines and vaccine-like monoclonal antibodies are currently ongoing but the development of a safe and effective vaccine for all at-risk populations remains challenging.^5^ Ribavirin is currently the only small molecule that has been approved for treatment of severe RSV infections by aerosol administration, but there is no clear proof of efficacy.^6^

In recent years, several direct-acting RSV inhibitors have entered clinical development, i.e. fusion inhibitors such as presatovir (GS-5806) and JNJ-678, and the lumicitabine (ALS-8176), a nucleoside inhibitor of the viral polymerase.^7^^,^^8^ Significant inhibition of RSV replication in human healthy volunteers experimentally challenged with RSV has been reported.^9^ Fusion inhibitors have a low barrier to resistance development; a single mutation in the viral target protein F compromises their antiviral activity. In addition, different classes of fusion inhibitors are typically cross-resistant.^10^ In contrast, the *in vitro* barrier to resistance to RSV nucleoside polymerase inhibitors (such as ALS-8176) has been shown to be very high and multiple mutations in the active site of the polymerase are required for the virus to acquire a resistant phenotype.^11^ Another promising RSV inhibitor in active preclinical development is PC786, a non-nucleoside inhibitor of RSV replication.^12^ Its exact mechanism of action is not fully understood, but it is clearly different from ALS-8176 as both classes differ chemically (non-nucleoside versus nucleoside) and no cross-resistance is observed.

The study of RSV antivirals has been standardized *in vitro* in cell lines, such as HEp-2 and HeLa, which permit reproducible assays at sufficient throughput. Here, we explore the use of fully differentiated human airway epithelium cells (HuAECs) of bronchial origin in an air–liquid interface to assess the efficacy of different classes of RSV inhibitors. This 3D culture system contains all relevant cell types of the lower respiratory tract (ciliated cells, goblet cells, mucus-producing cells) except for cells of the immune system. This system proved valuable in the study of infections with RSV and other respiratory virus infections.^13^

## Materials and methods

### Media, cells, virus and compounds

DMEM (catalogue no. 41965-039), PBS (catalogue no. 14190-094) and non-essential amino acid solution (NEAA; catalogue no. 11140-035) were obtained from Thermo Fisher Scientific. FBS was obtained from Hyclone (catalogue no. SV30160.03) and heat inactivated at 56°C for 30 min. HEp-2 cells and RSV-A Long strain were obtained from ATCC (catalogue no. CCL-23 and VR-26, respectively). HuAECs of bronchial origin in an air–liquid interface cell culture system and MucilAir medium were obtained from Epithelix (catalogue no. EP01MD and EP04MM, respectively), a Swiss company that has developed a technology that allows epithelium to reach a homeostatic state with a slow turnover. It is a ready-to-use product and commercially available (http://www.epithelix.com/). ALS-8112 and ALS-8176,^11^ AZ-27^14^ and PC786^15^ were synthesized according to published procedures (purity >98%). GS-5806 and TMC353121 were obtained from MedChemExpress (NJ, USA) and ribavirin was from ICN Pharmaceuticals (CA, USA).

### Virus and cells for in vitro standard antiviral assay

HEp2 cells were seeded at 5 × 10^3^ cells/well in a 96-well plate and were further cultured in DMEM (supplemented with 2% FBS and 1% NEAA) at 37°C and 5% CO_2_. The day after, medium was replaced by a serial dilution (1:2) of the antiviral test compounds in the same medium and the cultures were infected with RSV-A Long strain at an moi of 0.01 CCID_50_/cell (where CCID_50_ represents the 50% cell culture infectious dose in HEp2 cells). After 5 days, the typical cytopathic effect (CPE) was scored microscopically in all wells, on a scale of 0–5. The EC_50_ was calculated by logarithmic interpolation as the concentration of compound that resulted in a 50% protective effect against virus-induced CPE. Potential compound toxicity was evaluated in a similar set-up in treated, uninfected cultures where metabolic activity was quantified at day 5 by using the 3-(4,5-dimethylthiazol-2-yl)-5-(3-carboxymethoxyphenyl)-2-(4-sulfophenyl)-2H-tetrazolium (MTS) readout. The CC_50_ was calculated by logarithmic interpolation as the concentration of compound that resulted in a 50% decrease in the MTS signal.

### Infection of HuAEC inserts

HuAECs of bronchial origin from healthy donors were provided in porous culture inserts on an air–liquid interphase set-up and were maintained with MucilAir medium at 37°C and 5% CO_2_ for at least 4 days before use. Each insert contained 4 × 10^5^ cells (epithelix.com). An apical wash with PBS (300 μL) was performed just before infection with RSV-A Long strain (10 CCID_50_/insert). After 2 h, the inoculum was removed and an apical wash with PBS was collected (T0 of infection). Medium in the basal compartment of the HuAECs (basal medium) was refreshed concomitantly. Apical washes were collected at the indicated times post-infection and stored at −20°C until RNA extraction or at −80°C in culture medium supplemented with 20% sucrose for long-term storage. Antiviral compounds were added to the basal medium and refreshed daily for the duration of the treatment window at the indicated concentrations according to three regimens: (i) prophylactic treatment, in which the antiviral compound was administered 2 h before infection until day 4 post-infection; (ii) early therapeutic treatment, in which the compound was added from day 1 to day 5 post-infection; and (iii) late therapeutic treatment, in which the antiviral compound was added from day 3 until day 7 post-infection. For untreated cultures, the same concentration of DMSO as in the drug-treated inserts was added to the culture medium. Each condition tested was performed in duplicate.

### RNA extraction and RT-qPCR

Apical washes collected in PBS were subjected to RNA extraction using the NucleoSpin kit (Macherey-Nagel) according to the manufacturer’s instructions. Viral RNA was quantified by means of RT–qPCR with the iTaq Universal One Step Kit (Bio-Rad) on a Lightcycler 96 (Roche) thermocycler. RSV-A primers and probe for amplification were previously described.^16^ Genome copy values were calculated by using a standard curve obtained with increasing dilutions of a synthetic GeneBlock (IDT Technologies), corresponding to the sequence of the amplicon. Data were plotted with GraphPad software.

### Measurement of ciliary beat frequency (CBF)

At 4, 10 and 15 days post-infection, inserts were transferred in a six-well plate with a small meniscus of medium. An inverted microscope (Leitz, Labovert FS) was used at a magniﬁcation of ×600 to acquire images by a MotionScope high-speed camera at a temperature of 22°C. Images (at least 1024 images) were captured at 512 frames/s in at least three different areas of the insert. The CBF value was computed using custom-made Matlab software previously described.^17^^,^^18^ Briefly, the region of interest (ROI) was deﬁned as all pixels with signiﬁcant motion. Then, CBF values were calculated for each individual pixel in the ROI by fast Fourier analysis and expressed as a histogram of CBF values for all the pixels in the ROI. The mean CBF value of this histogram was used as the result for one CBF measurement. The average of at least three measurements per insert, i.e. six measurements per condition, is reported.

### RANTES mRNA level determination by RT-qPCR

HuAECs were harvested and cellular RNA was extracted with an RNA extraction kit (RNeasy Mini Kit, Qiagen). Specific primers for RANTES were designed and β-actin was used as an internal control. Samples were quantified by qPCR with the iTaq Universal Sybr Green One Step Kit (Bio-Rad) on a Lightcycler 96 (Roche) thermocycler. Fold change from the uninfected, untreated control was calculated according to the ΔΔCt method.

## Results

### Anti-RSV activity of selected reference antivirals in HEp-2 cells

The *in vitro* anti-RSV activity of a selection of reference compounds was first assessed in a CPE-based antiviral assay in HEp-2 cells in order to determine a common administration dose for the HuAEC system (Table 1). The entry/fusion inhibitors GS-5806^19^ and TMC353121,^20^ the nucleoside viral polymerase inhibitor ALS-8112 and its prodrug ALS-8176,^11^ the two non-nucleoside replication inhibitors AZ-27^21^ and PC786^12^ and the pan-antiviral ribavirin were included in the study. Fusion inhibitors elicited the most potent *in vitro* anti-RSV activity, followed by the non-nucleoside inhibitors and the nucleoside RSV inhibitors. Toxicity was evaluated in parallel and all the direct-acting anti-RSV compounds showed CC_50_ values in a concentration range at least 2 log_10_ higher than the EC_50_, highlighting a good selectivity index. Ribavirin proved the least active compound, with significant cytotoxicity in HEp-2 cells.

**Table 1.** Antiviral activity of reference compounds in HEp2 cells

	EC_50_^a^ (nM)		CC_50_^b^ (nM)	
	median (IQR)	number of replicates	median (IQR)	number of replicates
GS-5806	0.31 (0.20–0.51)	12	>100	14
TMC353121	0.46 (0.28–0.66)	65	>100	110
ALS-8176	470 (130–1300)	10	>50 000	18
ALS-8112	1300 (650–2000)	25	>50 000	8
Ribavirin	11 000 (8900–15 000)	164	42 000 (33 000–47 000)	70
AZ-27	21 (14–39)	6	>50 000	9
PC786	1.2 (0.81–1.3)	11	>50 000	13

a CPE scoring in RSV/HEp2 cell culture system.

b Toxicity in HEp2 cells using viability staining with MTS.

### Fusion inhibitors exert antiviral activity against RSV-infected HuAECs only with a prophylactic regimen

**Figure 1.**
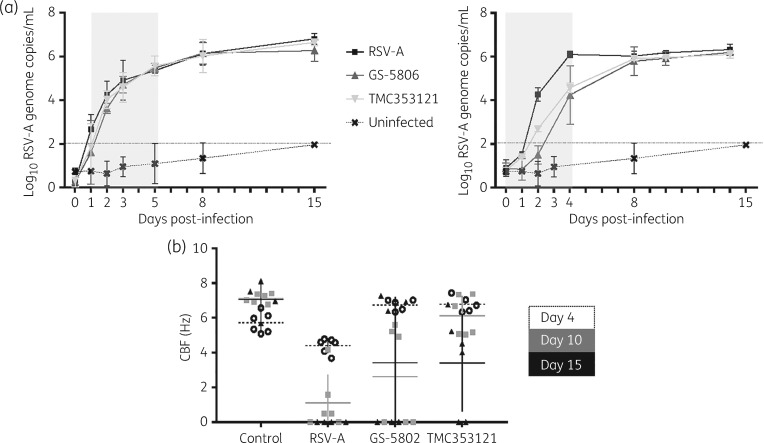
The effect of the RSV fusion inhibitors GS-5806 and TMC353121 was evaluated in HuAEC by using an early therapeutic treatment regimen (days 1–5 post-infection) (a, left panel) or by using a prophylactic regimen (−2 h to day 4 post-infection) (a, right panel). Compounds were added to the basal medium at a concentration of 100-fold the *in vitro* EC_50_. Apical washes were collected at selected timepoints post-infection and viral RNA was quantified by means of RT-qPCR. The treatment period is depicted in grey. The sensitivity threshold was determined by the highest RSV RNA signal quantified on uninfected, untreated cells (uninfected). Error bars represent the standard deviation of duplicates. (b) Ciliary beat frequency (CBF) was measured by high-speed video microscopy with a custom-made Mathlab script. Three areas per insert, i.e. six measurements per condition, were acquired at three data points, i.e. days 4–10 and day 15 post-infection. Average measurements per condition are represented as horizontal bars.

HuAEC inserts were infected with RSV-A (Long strain) and after 1 day (early treatment) or 3 days (late treatment) the fusion inhibitors GS-5806 and TMC353121 were added to the medium of the basal compartment at a concentration of 100-fold their EC_50_ in HEp-2 cells. RSV RNA levels were quantified in the apical washes at the indicated times post-infection. No antiviral effect was observed with either the early treatment (Figure 1a, left panel) or the late treatment (data not shown). By contrast, when GS-5806 (1000-fold *in vitro* EC_50_) and TMC353121 (100-fold *in vitro* EC_50_) were added 2 h before infection, a significant reduction in viral RNA levels was detected (>2 log_10_) by day 4 post-infection (Figure 1a, right panel). However, upon removal of the inhibitor from the culture medium, viral rebound was observed.

Infection of the cultures with RSV did not result in any noticeable cytopathic effect; therefore, the quantification of the CBF was used (in addition to quantifying of viral genomes levels) to assess the impact of the compound treatment on viral infection. Ciliary motility was quantified for at least three different areas per insert, i.e. at least six measurements per condition (Figure 1b). In line with an earlier report,^20^ a slight change in CBF was observed at day 4 post-infection, with 4.41 ± 0.39 Hz in infected, untreated versus 5.71 ± 0.54 Hz in uninfected inserts. By day 10 post-infection, all the analysed areas of RSV-infected untreated cultures showed complete dyskinesia (CBF 1.1 ± 1.4 Hz). In infected cultures, the treatment with fusion inhibitors delayed ciliary dyskinesia and in some of the areas of the drug-treated HuAEC cultures a CBF of 5–6 Hz was still noted.

### Polymerase inhibitors, nucleoside and non-nucleoside, show anti-RSV activity in a therapeutic set-up

**Figure 2.**
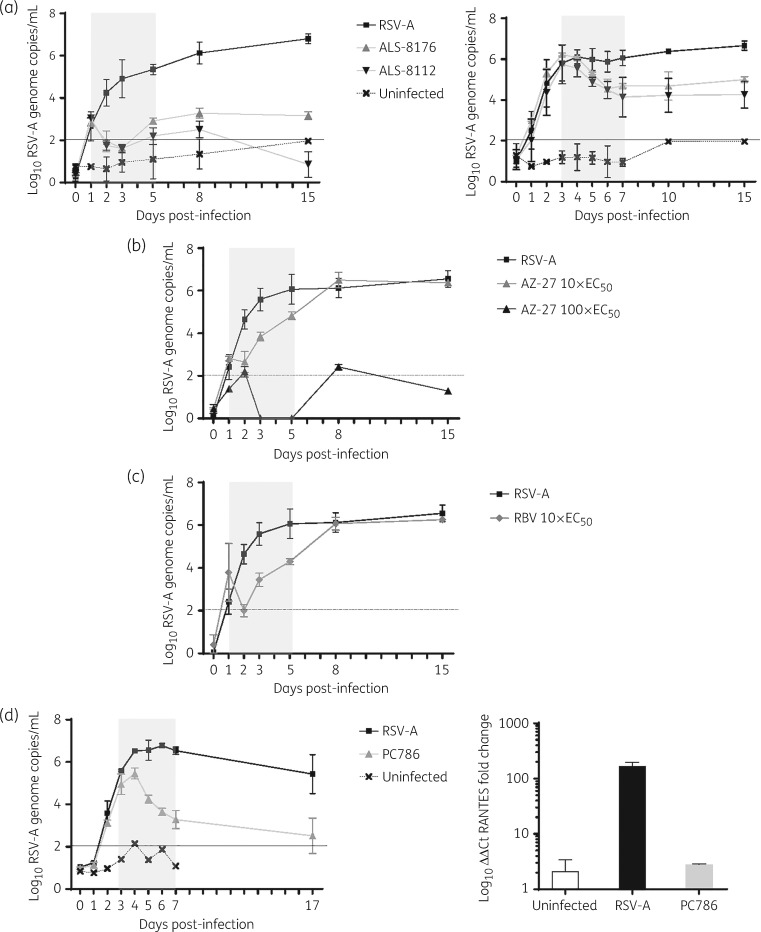
HuAEC were infected with RSV-A (Long strain) and were treated with: (a) the nucleoside viral polymerase inhibitor ALS-8112 or its prodrug ALS-8176 at 100-fold the *in vitro* EC_50_ by using either a late (days 3–7 post-infection, right panel) or early (days 1–5 post-infection, left panel) therapeutic treatment regimen; or (b) the replication inhibitor AZ-27 at concentrations 10- and 100-fold the *in vitro* EC_50_; or (c) ribavirin at 10-fold the *in vitro* EC_50_ with an early therapeutic treatment regimen; or (d) (left panel) the replication inhibitor PC786 at 100-fold the *in vitro* EC_50_ by using a late therapeutic treatment regimen. The treatment period is depicted in grey. The sensitivity threshold was determined by the highest RSV-RNA signal quantified on uninfected, untreated cells (uninfected). Error bars represent the standard deviation of duplicates. HuAEC were harvested at day 24 post-infection and total cellular mRNA was extracted. Levels of RANTES mRNA were quantified by means of RT-qPCR (d, right panel). β-actin was used as an internal control. Fold change of the uninfected, untreated control was calculated with the ΔΔCt method. Error bars represent the median of three independent quantifications of the condition and each condition was performed in duplicate (i.e. six measurements).

The potential anti-RSV activity of ALS-8112 and its prodrug ALS-8176 (both at 100-fold their EC_50_) was assessed in HuAECs with an early (day 1 to day 5 post-infection) and late (day 3 to day 7) therapeutic treatment. The early treatment resulted in a drop in viral RNA levels to (or lower than) the levels of the uninfected, untreated control (sensitivity threshold) and remained nearly undetectable, with a 4- and 6-log_10_ yield reduction by the end of the experiment (day 15 post-infection) for ALS-8176 and ALS-8112, respectively (Figure 2a, left panel). When the compounds were first added to the inserts at day 3 post-infection—when viral RNA levels had reached the plateau phase—a marked reduction (∼2 log_10_) in viral RNA was observed by day 7 post-infection. After the end of treatment, no viral rebound was detected (Figure 2a, right panel). The prodrug ALS-8176 was slightly less potent than the parental compound.

Next, the potential anti-RSV activity of the non-nucleoside replication inhibitor AZ-27 was assessed with an early therapeutic treatment regimen at concentrations of 10- and 100-fold the *in vitro* EC_50_. At 10-fold the EC_50_, a rapid decrease in viral RNA was observed after the start of treatment, followed by an equally rapid viral rebound after cessation of treatment (Figure 2b). At 100-fold the *in vitro* EC_50_, the effect of AZ-27 proved comparable to that of ALS-8112, with no detectable viral RNA on days 1–2 after the start of treatment and no viral rebound after stopping treatment (Figure 2b). Interestingly, like AZ-27 treatment at 10-fold the EC_50_, ribavirin at 10-fold the EC_50_ also resulted in a transient, sub-optimal antiviral effect (Figure 2c).

Another non-nucleoside replication inhibitor, PC786 (at 100-fold the EC_50_), resulted in the most pronounced antiviral activity, with a rapid decline in viral RNA yield in the apical washes even if the compound was first added to the inserts 3 days after infection. At the end of treatment (at day 7 post-infection), a 3 log_10_ reduction in viral RNA was noted and no rebound of viral replication was observed (Figure 2d, left panel). To explore whether antiviral treatment affected virus-induced immune activation of the HuAEC system, mRNA levels of the cytokine RANTES were quantified. This cytokine has been reported to be highly induced following RSV infection.^22^ RANTES mRNA levels were ∼200-fold higher in infected, untreated versus uninfected cultures (Figure 2d, right panel). Notably, in PC786-treated infected cultures, RANTES mRNA levels were comparable to those of the uninfected, untreated controls.

## Discussion

We present a model comprising reconstituted HuAECs of bronchial origin to study the efficacy of small-molecule inhibitors of RSV infection in a physiologically relevant context. To validate this model for antiviral studies, we explored the effect of a panel of RSV inhibitors in (pre-)clinical development targeting either viral fusion/entry (TMC35112 and GS-5806) or viral replication (the nucleoside ALS-8176 and the non-nucleosides AZ-27 and PC786).

For fusion inhibitors, an antiviral effect was only noted when the compounds were first added before the infection. Under these conditions, GS-5806 and TMC353121, regardless of the tested concentration (100- and 1000-fold the *in vitro* EC_50_, respectively), inhibited viral replication by >1 log_10_ (>90%) during the treatment window; however, a viral rebound was detected after stopping treatment. Possibly, entry inhibitors prevent only the first round of infection, but once the virus has entered the cell and spreads from cell to cell compounds of this class are no longer able to exert an antiviral effect. In a very recent study, the antiviral effect of another potent fusion inhibitor, JNJ-53718678, was explored in HuAECs.^23^ As in our study, the compound proved active when added prophylactically (1 h before infection). However, in apparent contrast with our findings, some (weak) activity of the JNJ fusion inhibitor was also detected in a therapeutic regimen (when the compound was added 1 day post-infection). Notably, the JNJ fusion inhibitor was added to both the basal and the apical compartment of the HuAEC inserts and the experimental endpoint was set at day 4 post-infection (the day of end of treatment). In our study, the inhibitor was only added to the basal compartment and viral replication was monitored daily until day 15 post-infection (i.e. 11 days after stopping treatment). The administration of the inhibitor to only the basal compartment might have affected the efficacy (because the infection occurs in the apical compartment) but might better reflect the situation in the infected patient receiving systemic dosing of the drug. It is noteworthy that in the first GS-5806 clinical trial on 37 healthy adults with low serum RSV RNA, the compound was administered orally following RSV detection but before symptom development.^24^ A recent mathematical modelling of the effect of fusion inhibitors on RSV treatment also suggests, in line with our findings, that the activity of fusion inhibitors strongly depends on early treatment.^25^ Despite the lack of an obvious clearance of RSV RNA, treatment with fusion inhibitors delayed the onset of ciliary dyskinesia. By means of high-speed video-microscopy, complete ciliary dyskinesia was observed by day 10 post-infection in RSV-infected HuAECs, whereas some ciliary beat activity in GS-5806- and TMC353121-treated samples was still noted at day 15 post-infection. Hence, treatment with fusion inhibitors, even if sub-optimal, may delay the onset of symptoms and be beneficial in natural infections, particularly for high-risk patients.

ALS-8112 is a cytidine nucleoside analogue and its prodrug, ALS-8176, is currently being evaluated in clinical trials (clinicaltrials.gov). In a human RSV challenge experiment, ALS-8176 reduced the absolute RSV viral load to undetectable levels in 1.3–2.3 days.^7^ In the HuAEC system, the compound was tested in an early therapeutic treatment and resulted in a marked antiviral effect. Similarly to the dynamics of activity observed *in vivo*, the virus became undetectable between day 1 and day 2 post-infection. A significant antiviral effect was also observed in the late therapeutic treatment schedule.

Overall, treatment with non-nucleoside replication inhibitors was effective when given early or late after infection, at least when concentrations ≥100-fold the *in vitro* EC_50_ were used. Indeed, lower concentrations (10-fold *in vitro* EC_50_) resulted in little effect and in an immediate rebound of viral replication after stopping treatment. Interestingly, no drug-resistant variants were selected following sub-optimal dosing of AZ-27. Among all the inhibitors studied, PC786 elicited the most potent activity in the HuAEC model; in particular it still resulted in a profound antiviral effect when first added to the infected cultures 3 days post-infection. In clinical practice early treatment is very difficult to realize, as patients most often seek medical advice 3–4 days post-infection. Therefore, drugs that are able to curb viral replication when added late after infection may likely hold most promise as therapeutic agents. Of clinical interest, ribavirin, the only antiviral approved for the treatment of RSV in high-risk populations but debated in terms of efficacy, proved inefficient in the control of RSV infection after stopping treatment in our model. Notably, in the clinic the recommended ribavirin treatment is oral, whereas in our model we mimicked a systemic treatment. There remains the need to address whether an apical administration of ribavirin in HuAECs will result in a persistent control of viral replication.

An added value of the HuAEC model is the possibility of studying the inflammatory response of a differentiated epithelium to viral infection. By monitoring inflammation markers, the efficacy of an antiviral treatment in curing the system and restoring its innate immune state can be studied. We monitored (mRNA levels of) RANTES, a cytokine highly expressed during RSV infection, and noted a ∼200× induction upon infection. Treatment with PC786 completely abrogated this increase, indicating that RANTES is a good marker of RSV infection in HuAECs and suggesting that effective antiviral treatment might restore the innate immune state of the system.

In conclusion, we describe a robust and reproducible model to study RSV infection and to assess the potency and the antiviral dynamics of RSV inhibitors. 3D *ex vivo* cultures are widely debated for their clinical relevance as surrogates of human infection. We demonstrate that RSV-infected 3D cultures of HuAECs recapitulate aspects of the response to antiviral therapy and provide further evidence that the use of HuAECs is a relevant cell-based model for disease and antiviral studies.
